# Effectiveness of endoscopic intranasal incision reduction for nasal fractures

**DOI:** 10.1007/s00405-021-06878-3

**Published:** 2021-05-24

**Authors:** Shu Yan, Yan Jiang, Yan Wang, Kaixuan Chen, Xudong Yan, Xiaohan Sun, Jisheng Zhang, Na Li

**Affiliations:** Department of Otolaryngology-Head and Neck Surgery, Medical Research Center, The Affiliated Hospital of Qingdao University, Qingdao, 266003 China

**Keywords:** Nasal bone fractures, Fractures of the frontal process of the maxilla, Nasoseptal fractures, Endoscopic intranasal incision reduction, Aesthetics

## Abstract

**Purpose:**

To report our experience using endoscopic intranasal incision reduction (EIIR) for nasal fractures and to assess effectiveness of the method.

**Methods:**

30 patients who underwent EIIR were retrospectively analysed. All the patients were examined by three-dimensional computed tomography (3D CT), acoustic rhinometry and rhinomanometry, preoperatively and postoperatively at 1 month. The visual analogue scale (VAS) was used to assess the preoperative aesthetics and nasal airflow satisfaction and at 1, 3 and 6 months postoperatively. VAS aesthetic satisfaction was also scored by two junior doctors.

**Results:**

3D CT showed that the fracture fragments fitted well in 30 patients postoperatively at 1 month. VAS aesthetics and nasal airflow scores were significantly improved postoperatively at 1, 3 and 6 months compared with preoperative scores (*P* < 0.01). The VAS aesthetic scores from the two surgeons were also significantly improved (*P* < 0.01). The minimal cross-sectional area increased from 0.39 ± 0.13 to 0.64 ± 0.13 (*P* < 0.001), the nasal volume increased from 4.65 ± 0.86 to 6.37 ± 0.94 (*P* < 0.001) and the total inspiratory airway resistance of the bilateral nasal cavity median decreased from 0.467 Pa/mL/s to 0.193 Pa/mL/s (*P* < 0.001). There were no technique-related intraoperative complications.

**Conclusion:**

EIIR was a practical choice, and the aesthetics and nasal airflow were significantly improved in patients with overlapped and displaced bone fragments, patients with fractures of the frontal process of the maxilla (FFPM), patients who underwent failed CR and patients beyond the optimal temporal window.

## Introduction

The bony framework of the nose is mainly composed of the nasal bones, the frontal process of the maxilla (FPM) and the nasal process of the frontal bone. Nasal bones affect the height of the nasal bridge, and FPM determine the width of the nasal dorsum, whilst the nasal septum supports nasal bones. It has been reported that nasal fractures account for greater than 37% of post-traumatic maxillofacial fractures, which is the most common facial traumatic fracture [1]. Nasal fractures often lead to external nasal deformities and dysfunctional nasal airflow. Nasal fractures may result in profound psychological and functional impacts owing to abrupt changes in physical appearance accompanied by nasal obstruction [2].

The main methods for treating nasal fractures include closed and open reduction. The closed reduction (CR) method was more widely applied. Although CR is a simple, fast and economical method, the procedure is performed blindly and is mainly guided by the experience of the surgeon. The outcomes of CR vary widely throughout the literature with patient satisfaction rates being reported between 30% [3, 4] and 95% [5]. Some studies reported revision surgery rates after CR were between 5.5% and 11% [6–11].

Indirect open reduction of nasal fractures was first introduced by Burm and Oh using an endonasal incisional approach in 1998 [12]. Since then, the technique has been reported by many surgeons and shown to be more efficient than CR [13]. However, it has a limited field of view and submucoperiosteal dissection may damage the blood supply causing cartilage collapse or irregularities in the nose [14]. Since direct observation for some subjects of nasal fractures is crucial, the introduction of endoscope and the position of incision seem promising for adequate treatment and optimal outcomes. Now we introduce the endoscopic intranasal incision reduction (EIIR) technique as an alternative approach for nasal fractures, additionally present our experience and demonstrate the efficacy of the technique.

## Methods

### Patients

**Fig.1:**
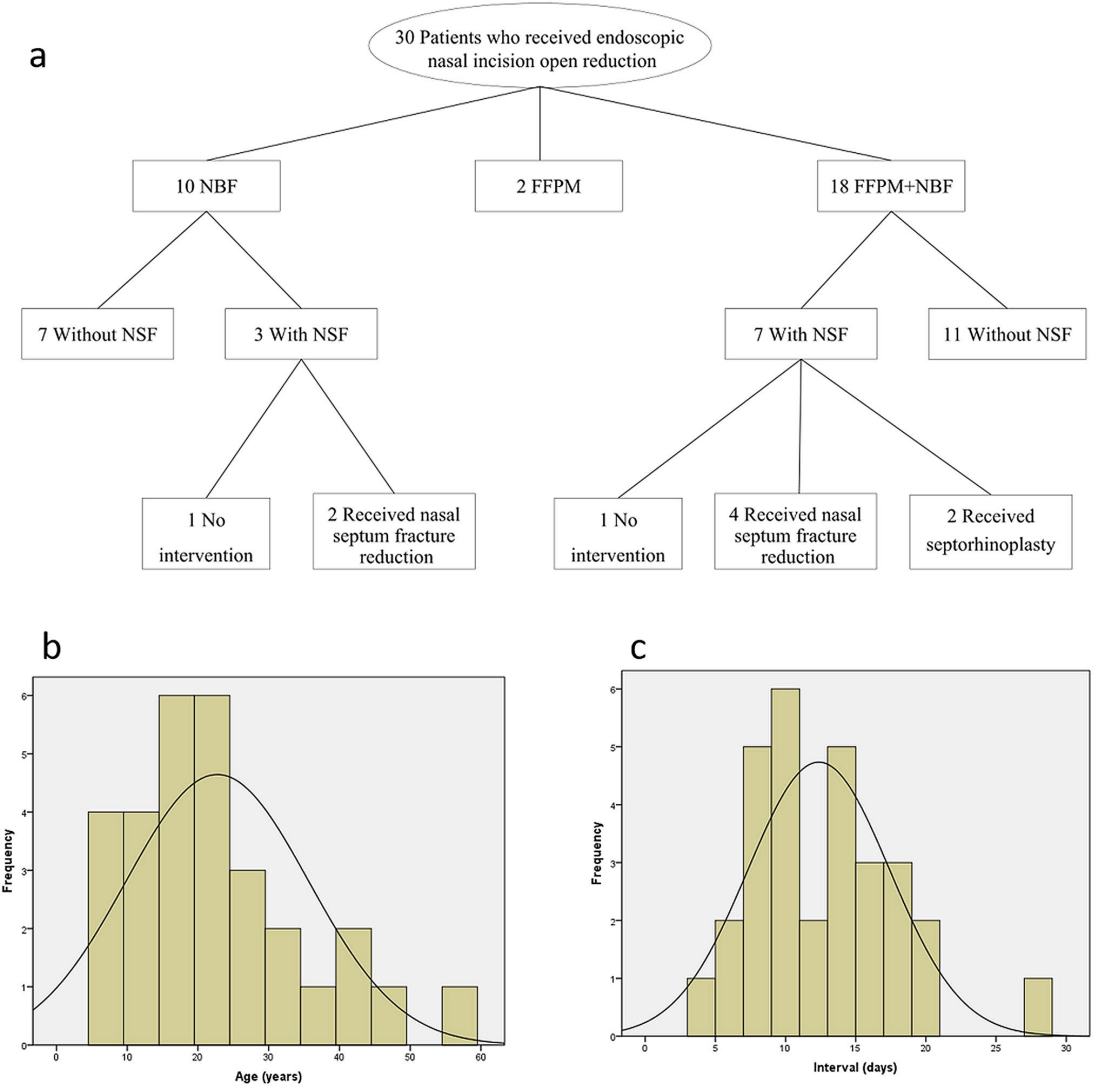
Demographic and clinical characteristics of the patients with nasal fractures. **a** Kinds of trauma and operation method of the 30 patients with nasal fractures. **b** Distribution of age, median age (P25,P75) is 20.50 (12.50, 29.25). **c** Distribution of trauma time, median trauma time (P25,P75) is 11.00 (8.00,16.00). NBF the nasal bone fractures, FFPM fractures of the frontal process of the maxilla, NSF nasal septum fractures

Retrospective review was conducted with 30 patients of nasal fractures who underwent EIIR in the Affiliated Hospital of Qingdao University between August 2018 and May 2020 (Fig. 1a). The cohort comprised 23 males and 7 females aged between 7 and 57 (22.73 ± 12.89) years (Fig. 1b). The patients were underwent surgery at 4–27 (12.37 ± 5.06) days after trauma (Fig. 1c). 10 patients were combined with nasoseptal fractures (NSF). 5 patients underwent failed CR. All patients were followed up at least 6 months after surgery.

Inclusion criteria: (1) Nasal bone three-dimensional computed tomography (3D CT) showing nasal fractures with displaced fragments, with/without NSF, including unilateral and bilateral fractures. It means including severe fractures of type II and mild fractures of type III according to Hwang classification [15]; (2) Trauma time within 4 weeks; (3) Patients who underwent failed CR within 2 weeks; (4) Patients aged between 6 and 60 years who had finished follow-up at 1, 3 and 6 months postoperatively.

Exclusion criteria: (1) Patients who had received surgery on the external nose, nasal cavity or sinus within 6 months; (2) Patients suffering from mental illnesses.

### Study design

All the patients were examined by 2 mm 3D CT of the nasal bone before surgery and at 1 month after operation to evaluate fracture reduction. 3D CT scans were reviewed by an independent radiologist blinded to the goals of the study. All patients were evaluated to check the status of the nasal septum and nasal cavity using nasal endoscope preoperatively and at 1, 3 and 6 months postoperatively.

The visual analogue scale (VAS) was used for aesthetic and nasal airflow satisfaction ranging from 1 to 10 (10 points representing the highest satisfaction) [16]. Each patient scored their aesthetic and nasal airflow satisfaction preoperatively and at 1, 3 and 6 months postoperatively. The VAS of aesthetic satisfaction were also independently scored by two junior doctors who had not participated in the operations and had no conflict of interest with the surgeon. All the operations were performed by the same senior doctor.

The main nasal airflow parameters included minimal cross-sectional area (MCA), 0–6 cm nasal volume (NV), total inspiratory airway resistance of bilateral nasal cavity (AR) that were measured before and one month after surgery based on acoustic rhinometry and rhinomanometry (manufactured by GM Instruments Ltd) to provide objective insights into nasal airflow [16].

### Statistical analysis

Statistical analyses were performed using SPSS 25.0 software. A paired *t* test was used to analyse differences in MCA and NV before and after the operations. Differences between the pre- and postoperative AR were analysed using a signed rank-sum test. VAS scores before the operations were compared with those obtained at 1, 3 and 6 months after the operation using a signed rank-sum test. *P* < 0.05 indicates statistical significance.

### Surgical technique

**Fig.2:**
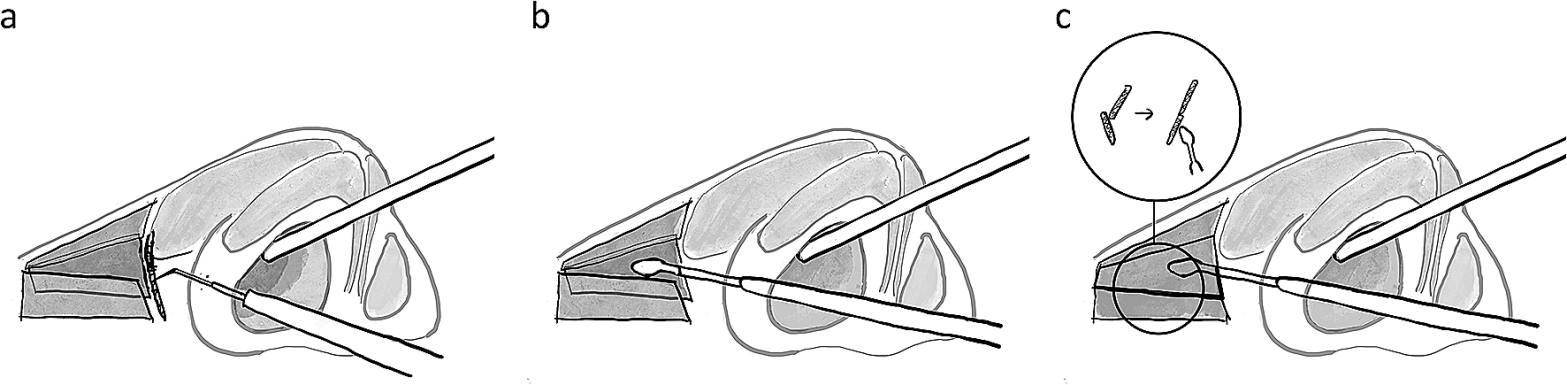
Schematic diagrams to display the technique of EIIR. **a** The incision at the rim of the piriform aperture with a stylet electric knife. **b** Separate and expose the displaced fracture fragments under the periosteum. **c** Fracture fragments were moved back into the position pre-trauma using an elevator

**Fig.3:**
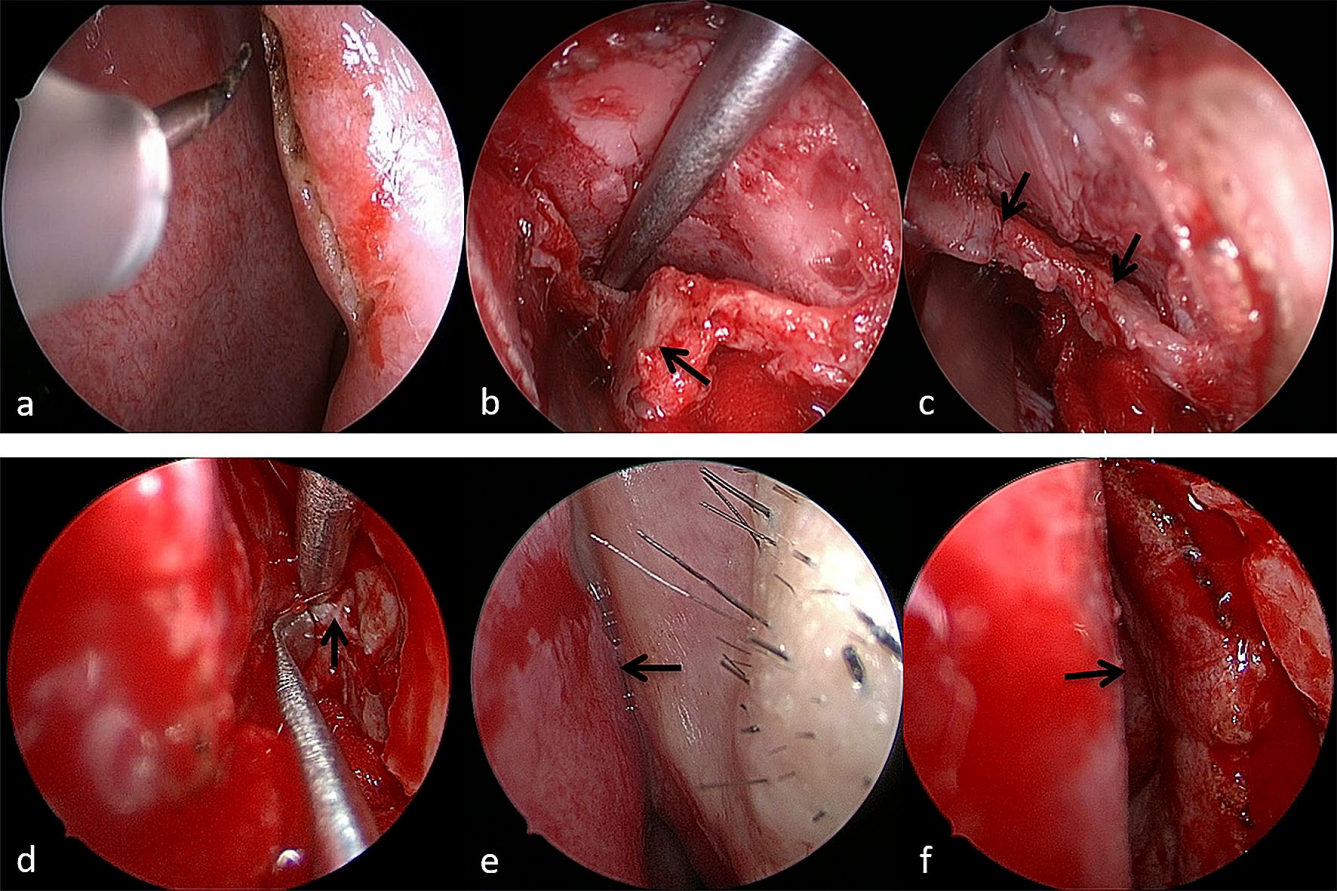
Surgical procedures of the EIIR. **a** The incision at the rim of the piriform aperture. **b** The displaced fracture FPM. **c** The fracture lines after reduction. **d** The soft tissue embedded among the bone fragments. **e** The narrow internal nasal valve caused by collapsed FPM. **f** The wide internal nasal valve after reduction

All operations were performed under general anaesthesia. The position of the incision was located at the rim of the piriform aperture on the lateral wall of the nasal cavity on the fracture site (Fig. 2). A 1 cm arc incision was made in the determined incision site using a nasal endoscope (Fig. 3a). The soft tissue was then cut from the mucosa to the periosteum with a stylet electric knife and separated from the soft tissue under the periosteum. The separation range extended beyond the upper edge of the fracture area, nearly reaching the nasal bone midline to the inside, and touching the external area of the FFPM. The area of the fracture-displacement was exposed and the dislocated nasal bone with/without the FPM was separated (Fig. 3b). In cases where the early callus had formed, it was removed by soft peeling. If some soft tissue are embedded among the bone fragments, we should release the embedded tissue completely (Fig. 3d).

Bone fragments were moved back into the position pre-trauma using an elevator (Fig. 3c). The results of the bone fragment matching were observed under a nasal endoscope. The surgeon was satisfied with the appearance of the nose and then the periosteum and mucosa of the incision were cut open. According to the fracture conditions, a unilateral or bilateral nasal incision was performed. A gelatin sponge was applied to the surface and an iodoform gauze placed into the top and side-walls of the nasal cavity to support the fracture area. Depending on the fracture conditions, the nasal packing was removed within 3–7 days and the external nose was fixed with a nasal splint for 3–7 days.

## Results

**Fig.4:**
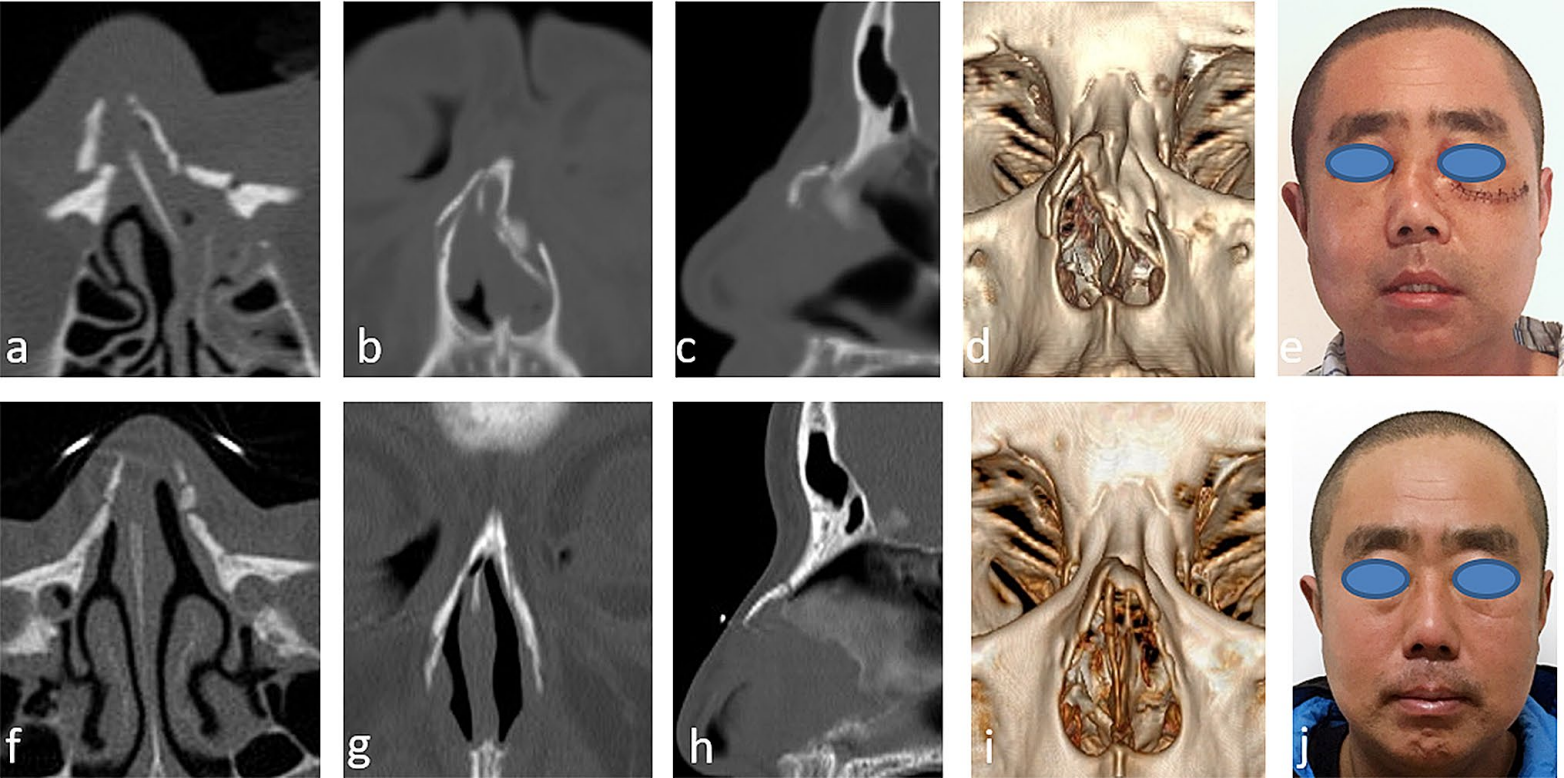
Images of a patient with nasal fractures. A 40-year-old male was knocked down by a heavy object resulting in nasal bridge deviation at work. After 7 days, he underwent EIIR and obtained satisfied aesthetics and good nasal airflow postoperatively. **a** Preoperative axial CT scan. **b** Preoperative coronal CT scan. **c** Preoperative sagittal CT scan. **d** Preoperative 3D CT scan. **e** Preoperative photo showing a depressed nasal bridge. **f** Postoperative axial CT scan. **g** Postoperative coronal CT scan. **h** Postoperative sagittal CT scan. **i** Postoperative 3D CT scan. **j** 6 month postoperative photo showing a well-corrected nasal bridge

Good aesthetic effects were achieved in 30 patients. Good nasal airflow on both sides was achieved in 28 patients. 2 patients were not satisfied enough with the improvement of nasal obstruction, but nasal obstruction improved after using intranasal glucocorticoid for 1 month and the patients did not demand secondary surgery. Among the 10 patients with NSF, 2 cases without nasal obstruction gave no intervention, 6 cases received nasal septum fracture CR, and 2 cases underwent septoplasty. The incisions had healed well at 1 month postoperatively. The nasal bone CTs showed good fracture union at 1 month postoperatively (Fig. 4a and f, b and g, c and h, d and i).

**Fig.5:**
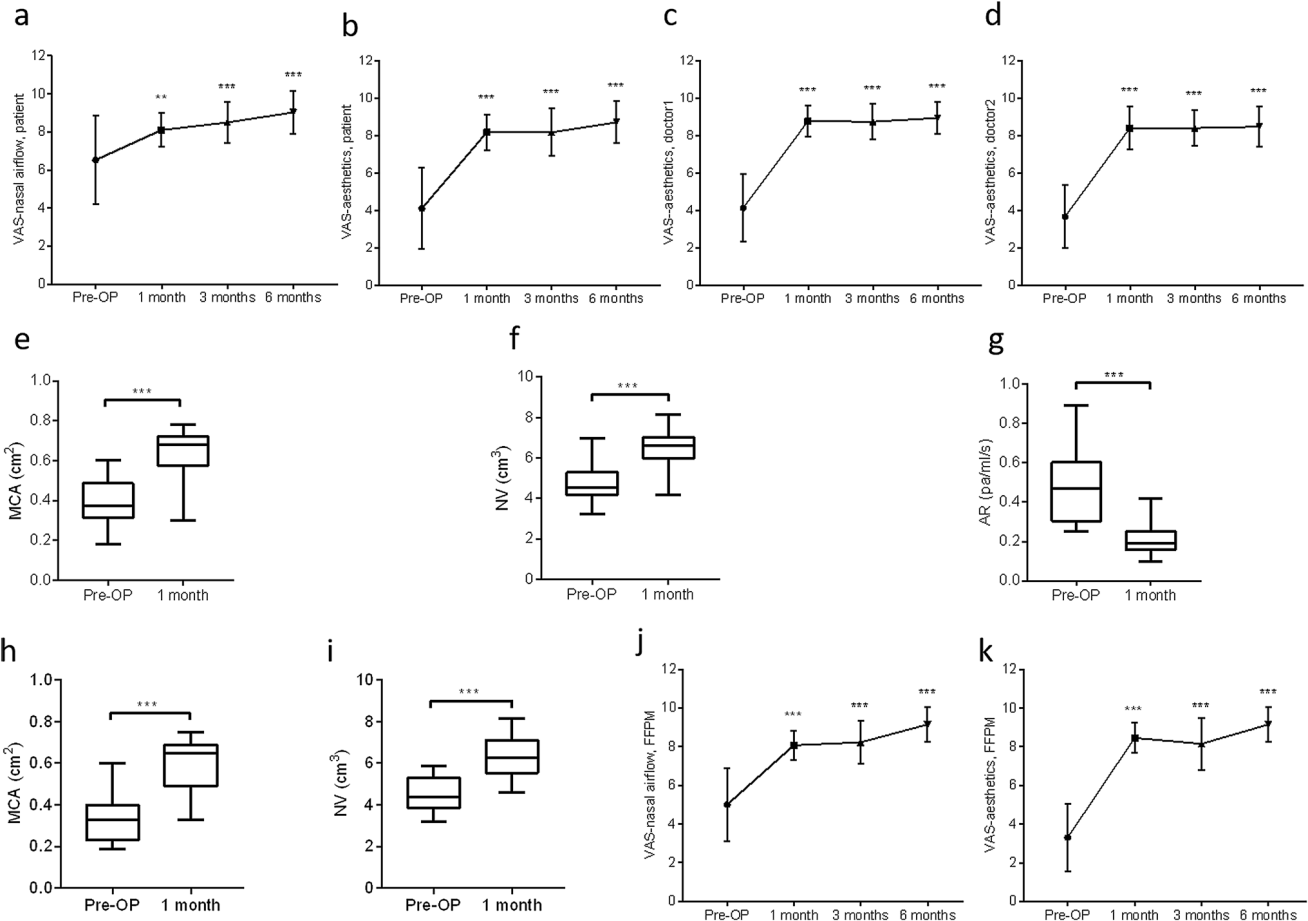
Difference of VAS scores, MCA, NV and AR postoperatively compared with preoperatively. **a** Difference of VAS scores of nasal airflow satisfaction reported by patients. **b** Difference of VAS scores of aesthetic satisfaction reported by patients. **c** Difference of VAS scores of aesthetic satisfaction reported by one junior doctor. **d** Difference of VAS scores of aesthetic satisfaction reported by another junior doctor. **e** Difference of MCA postoperatively (0.64 ± 0.13) compared with preoperatively (0.39 ± 0.13). **f** Difference of NV postoperatively (6.37 ± 0.9) compared with preoperatively (4.65 ± 0.86). **g** Difference of AR postoperatively (median 0.193) compared with preoperatively (median 0.467). **h** Difference of MCA postoperatively (0.59 ± 0.04) compared with preoperatively (0.33 ± 0.03) of 13 patients with FFPM. **i** Difference of NV postoperatively (6.34 ± 0.24) compared with preoperatively (4.37 ± 0.23) of 13 patients with FFPM. **j** Difference of VAS scores of nasal airflow satisfaction reported by 13 patients with FFPM. **k** Difference of VAS scores of aesthetic satisfaction reported by 13 patients with FFPM

VAS scores of nasal airflow of the patients (Fig. 5a) were significantly improved postoperatively at 1 (median 8.00), 3 (median 9.00) and 6 (median 9.00) months compared with preoperative (median 6.00) scores (*P* = 0.005). VAS scores of aesthetics of the patients (Fig. 5b) were significantly improved postoperatively at 1 (median 8.00), 3 (median 8.00) and 6 (median 9.00) months compared with preoperative (median 4.00) scores (*P* < 0.01). VAS scores of aesthetics of one doctor (Fig. 5c) were significantly improved postoperatively at 1 (median 9.00), 3 (median 9.00) and 6 (median 9.00) months compared with preoperative (median 4.00) scores (*P* < 0.01). VAS scores of aesthetics of another doctor (Fig. 5d) were significantly improved postoperatively at 1 (median 9.00), 3 (median 8.00) and 6(median 9.00) months compared with preoperative (median 4.00) scores (*P* < 0.01).

MCA (Fig. 5e) increased from 0.39 ± 0.13 cm^2^ to 0.64 ± 0.13 cm^2^, the NV (Fig. 5f) increased from 4.65 ± 0.86 cm^3^ to 6.37 ± 0.94 cm^3^ and the AR (Fig. 5g) median decreased from 0.467 Pa/mL/s to 0.193 Pa/mL/s at 1 month postoperatively compared with preoperatively.

For those 13 patients with FFPM, VAS scores of nasal airflow (Fig. 5j) were significantly improved postoperatively at 1 (median 8.00), 3 (median 9.00) and 6 (median 9.00) months compared with preoperative (median 6.00) scores (*P* = 0.001). VAS scores of aesthetics (Fig. 5k) were significantly improved postoperatively at 1 (median 8.00), 3 (median 8.00) and 6 (median 9.00) months compared with preoperative (median 3.00) scores (*P* < 0.01). MCA (Fig. 5h) increased from 0.33 ± 0.03 cm^2^ to 0.59 ± 0.04 cm^2^ (*P* < 0.001) at 1 month postoperatively compared with preoperatively. The NV (Fig. 5i) increased from 4.37 ± 0.23 cm^3^ to 6.34 ± 0.24 cm^3^ at 1 month postoperatively compared with preoperatively.

None of the patients had persistent deformities and none of them required revision surgery. One patient suffered from nasal bleeding after removing the packing and underwent subsequent electrocoagulation under surface anaesthesia. No other postoperative complications were observed during follow-up.

## Discussion

In general, CR is the most common treatment for nasal fractures due to its short surgery time and relatively low invasiveness [17]. However, CR can lead to unsatisfactory results, particularly in patients with FFPM. For patients who have missed the optimal time for the operation (within 2 weeks after trauma) [18], CR is complicated by the formation of the callus which affects the operation. A retrospective review of 607 patients with nasal fractures reported that although only 5% of patients were dissatisfied with CR, 35% had some degree of nasal obstruction and 9% had nasal deviation [5]. Studies noted that 11–50% of patients had persistent deformities after CR and needed subsequent septoplasty [6, 19].

Currently, another common treatment for nasal fractures is open reduction through an inter-cartilaginous incision under direct vision [13, 14]. Although this technique has certain advantages, it is not recommended for patients under 16 or in those with comminuted nasal fractures. This is because submucoperiosteal dissection at the interchondral incision may damage the development of the blood supply of the nasal bone and the resulting scar may cause cartilage collapse or irregularities in the nose [13]. The scar tissue at the inter-cartilaginous incision may influence the internal nasal valve and may affect nasal airflow. In our study, incision was at the rim of the piriform aperture which do not damage cartilage and more closer to the fracture site. The fracture site can be exposed directly and accurate reduction can be got with a nasal endoscope. Nasal endoscopy can extend the field of vision with small incision and video connection permits easy training of residents. In our experience, overlapped and misplaced bone fragments are difficult to replace unless separate it free. On the other hand, the soft tissue surrounding the fracture fragments also plays an important role. Some bone fragments are connected by fibrous tissues and some soft tissue are embedded among the bone fragments. Therefore, it is necessary to expose the broken ends and loosen the soft tissues for patients with overlapped and misplaced bone fragments, for patients with tissues stretched and embedded among the bone fragments. The EIIR technique could provide an effective choice for these subjects.

The optimal time for the reduction of the nasal bone fracture remains to be fully clarified [13]. The main point is that reduction should be performed 10–14 days after trauma [18]. However, in our study, all surgeries performed in 4 weeks after trauma obtained satisfactory results since the early callus could be removed by the elevator directly. EIIR provides an alternative option in the treatment of patients outside the optimal temporal window.

In patients with FFPM, the fracture site was located in the lateral nasal wall and obscured by the inferior turbinate. Instruments for CR were difficult to reach the fracture area due to the obstruction of the inferior turbinate, especially for overlapped and dislocated bone fragments. In our study, 5 patients who underwent failed preoperative CR were combined with FFPM. The method of EIIR made a small incision at the rim of the piriform aperture to expose the fracture site of the FPM directly. Then, it was easy to get accurate reduction. Nonetheless, attention should be paid to the tightness of the nasal packing and fixation to avoid a wide nasal dorsum. On the other hand, fracture and collapse of the MFP may affect the internal nasal valve which is a common cause of nasal obstruction (Fig. 3e, f). Nasal valve collapse can be innate to patient anatomy, iatrogenic, congenital, or traumatic [20]. EIIR was suitable for this kind of nasal fractures. In our study, VAS scores of nasal airflow and aesthetics, MCA and NV were significantly improved at 1 month postoperatively compared with preoperatively. However, the sample size was small, and a larger group with extended follow-up is required to verify the viewpoint.

Concurrently performing NSF can improve nasal airflow and also facilitate nasal bone reduction. In our study, amongst the 10 patients with NSF, 6 cases received closed nasal septum fracture reduction, 2 cases received septoplasty and the other 2 cases without nasal obstruction had no intervention. All of the 10 patients acquired good nasal airflow and aesthetics. In determining the optimum therapeutic regime in nasal bone fractures, prior nasal obstructions, nasal septum deviation or other aspects of the patient’s medical history should all be considered [21]. The following are the classical indications for nasoseptal surgery: (1) the presence of a septal hematoma; (2) a septal deviation with partial or total airway obstruction; and (3) bone or cartilaginous tissue severely damaged or protruding through the septal mucosa demonstrated clinically or radiologically [22]. Treatment of displaced septal fractures may be with a closed or an open approach. Endoscopic septoplasty is a widely described technique for the approach [23]. In this study, 1 patient received septoplasty who had nasal obstruction both before and after trauma. This patient was likely to have a deviated nasal septum before the trauma. The other patient who received septoplasty had fractures of both the septal cartilage and the lamina plate of the ethmoid bone and had an angular deformity. Fractured bones were opposed to one side of the inferior turbinate. This patient was also one of the 5 patients who underwent failed CR. NSF is usually related to the collapse and displacement of the nasal bone. This type of fracture requires treatment both of the nasal bone and septum at the same time.

Conclusion, EIIR was a practical choice, and the aesthetics and nasal airflow were significantly improved in patients with overlapped and displaced bone fragments, patients with FFPM, patients who underwent failed CR and patients beyond the optimal temporal window.

This study was just a single-center retrospective study with a small sample size, and a larger group with extended follow-up is required to verify the effectiveness of the technique.
